# Rapid Physicochemical Screening of Parboiled Rice Using VNIR–SWIR Spectroscopy and Chemometrics

**DOI:** 10.1111/1750-3841.71501

**Published:** 2026-09-30

**Authors:** Nairiane dos Santos Bilhalva, Marisa Menezes Leal, Rosana Santos de Moraes, Dthenifer Cordeiro Santana, Larissa Pereira Ribeiro Teodoro, Paulo Eduardo Teodoro, Paulo Carteri Coradi

**Affiliations:** ^1^ Laboratory of Postharvest (LAPOS), Campus Cachoeira do Sul Federal University of Santa Maria Cachoeira do Sul Rio Grande do Sul Brazil; ^2^ Campus de Chapadão do Sul Federal University of Mato Grosso do Sul Chapadão do Sul Mato Grosso do Sul Brazil

**Keywords:** indirect methods, parboiled rice, proximate composition, quality control, spectral preprocessing

## Abstract

Efficient quality assessment of parboiled rice is important for industrial processing, but conventional analytical procedures are often time‐consuming, reagent‐intensive, and destructive. This study evaluated VNIR–SWIR spectroscopy combined with chemometric modeling for the rapid prediction of moisture, starch, protein, lipids, fiber, and ash in parboiled rice representing commercial types from Type 1 to Type 5 and Off‐Type. Partial least squares regression models were developed using six spectral preprocessing strategies and evaluated through internal cross‐validation and a held‐out test set. Model performance varied substantially among the physicochemical attributes. Starch showed the best test‐set performance using baseline offset preprocessing (*R* ^2^ = 0.740; RMSEP = 0.902 percentage points), while moisture showed promising results using MSC, baseline offset, or untreated spectra (*R* ^2^ = 0.627; RMSEP < 0.37 percentage points). Ash showed moderate performance with SNV combined with baseline correction and Savitzky–Golay smoothing (*R* ^2^ = 0.492; RMSEP = 0.045 percentage points). In contrast, protein and fiber showed limited predictability, with maximum *R* ^2^ values of 0.112 and 0.248, respectively. SNV and MSC generally improved models affected by scattering, whereas baseline offset was most effective for starch. Overall, VNIR–SWIR spectroscopy showed potential for rapid screening, particularly for starch and moisture, although broader datasets and validation using independent production batches are required before routine industrial implementation.

## 1 Introduction

Parboiled rice is widely consumed and valued for its nutritional and technological properties resulting from parboiling, a hydrothermal process involving soaking, steaming, and drying. This treatment promotes starch gelatinization and retrogradation (Rodrigues et al. 2024), changes the starch granule structure from a crystalline to an amorphous state, enhances endosperm translucency, and reorganizes the molecular arrangement of starch (Müller et al. 2020). Parboiling may also increase iron retention through the migration of minerals from the outer grain layers into the endosperm (Taleon et al. 2020; Bagchi et al. 2023).

In addition to starch, moisture, protein, lipids, fiber, and ash are important indicators of rice quality. Moisture directly affects storage stability and shelf life; protein contributes to nutritional value; lipids influence energy density and oxidative stability; fiber is associated with nutritional and digestive properties; and ash reflects the mineral composition of the grain. The combined assessment of these attributes supports quality characterization, processing decisions, storage management, and product differentiation (Bilhalva et al. 2025a; Bilhalva et al. 2025b; Anjum et al. 2025; Carneiro et al. 2025).

Accurate characterization of the physicochemical properties of parboiled rice, particularly its proximate composition, is essential for ensuring product quality and supporting industrial decision‐making (Coradi et al. 2021). However, conventional analytical methods may require chemical reagents, trained personnel, long execution times, and sample destruction. In this context, reflectance spectroscopy in the visible, near‐infrared (NIR), and short‐wave infrared regions has emerged as a rapid and non‐destructive alternative capable of evaluating multiple quality attributes from a single spectral measurement (Hayati et al. 2021; Arf et al. 2026).

Recent advances in broadband optical detection have also contributed to the development of spectral sensing technologies. Li et al. (2023) developed a high‐performance UV–Vis–NIR photodetector with an extended spectral response. Although this device was not developed specifically for food analysis and differs from the reflectance spectroradiometer used in the present study, it provides relevant context regarding the continuing development of broadband optical detection systems.

VNIR–SWIR reflectance spectroscopy evaluates grain quality based on the interaction between electromagnetic radiation and the physical and chemical characteristics of the sample. In the present study, spectral data were initially acquired from 350 to 2500 nm. The visible region, approximately 350–750 nm, may provide information related to grain color, surface characteristics, and visually detectable defects. However, this region was excluded from quantitative modeling because it presented higher spectral noise and limited relevant information for the physicochemical attributes evaluated. Therefore, the quantitative analyses were conducted using the NIR–SWIR interval, which contains broad and overlapping overtone and combination absorption bands associated mainly with O–H, C–H, and N–H molecular vibrations. These molecular groups are related to moisture, starch, protein, lipids, and other organic constituents of rice.

Depending on the instrument configuration, conventional NIR spectroscopy generally covers approximately 780–2500 nm and therefore substantially overlaps with the infrared portion of the VNIR–SWIR range. Conventional NIR spectroscopy is widely used for the rapid bulk analysis of moisture, protein, starch, and lipids. The VNIR–SWIR configuration provides broader initial spectral coverage by integrating visible, NIR, and short‐wave infrared information within the same acquisition. However, the most informative wavelength interval must be selected according to the analytical objective, the physicochemical attribute under investigation, and the quality of the acquired spectra.

Raman spectroscopy differs from NIR and VNIR–SWIR reflectance spectroscopy because it is based on inelastic light scattering and produces relatively narrow molecular fingerprint bands associated with specific vibrational modes. Raman spectroscopy is particularly useful for assessing molecular structures and transformations, such as starch gelatinization. In contrast, NIR and VNIR–SWIR spectroscopy are especially suitable for rapid bulk screening and the simultaneous evaluation of multiple physicochemical attributes. Nevertheless, these techniques should be considered complementary rather than directly interchangeable, because their performance depends on the target attribute, instrument configuration, sample characteristics, and modeling strategy.

The technology used in the present study must also be distinguished from hyperspectral imaging. Although the spectroradiometer records a large number of contiguous wavelength variables, it does not generate a spatially resolved hyperspectral image. Hyperspectral imaging produces a three‐dimensional data cube containing two spatial dimensions and one spectral dimension, providing an individual spectrum for each image pixel (Gao and Smith 2015) . In contrast, the FieldSpec system combined with the Muglight accessory records a non‐imaging spectrum representing the average optical response of the measured sample area.

Hyperspectral imaging is advantageous for applications requiring pixel‐level classification, visualization of chemical distributions, identification of individual defective grains, or mapping of sample heterogeneity. However, it generally produces larger datasets and requires more complex image processing and dimensionality‐reduction procedures. The non‐imaging approach used in the present study generates smaller datasets, requires less computational processing, and is suitable for rapid compositional screening of intact bulk samples. Its main limitation is that it cannot identify the spatial location of defective grains or visualize variations within individual grains.

Recent investigations have demonstrated the potential of NIR, VNIR–SWIR, and hyperspectral technologies for predicting rice quality, although model performance varies considerably among attributes and experimental conditions. Hayati et al. (2021) used NIR spectroscopy from 1000 to 2500 nm and PLS regression to predict rice protein content, obtaining a correlation coefficient of 0.90 and an RPD of 3.19. Song et al. (2023), using hyperspectral imaging from 900 to 1700 nm, variable selection, and PLS regression, obtained prediction coefficients of determination of 0.9650 and 0.9567 for moisture in paddy and polished rice, respectively. In contrast, the corresponding values for fatty acid content were lower, at 0.8573 and 0.8436, demonstrating that predictability depends strongly on the physicochemical attribute being evaluated.

For parboiled rice, Taghinezhad et al. (2025) reported high predictive performance for starch gelatinization and head rice yield using Raman and NIR spectroscopy combined with multivariate and artificial neural network models. However, their models were developed for specific processing attributes under controlled soaking and drying conditions rather than for the simultaneous prediction of proximate composition across different commercial types.

Using non‐imaging VNIR–SWIR spectra acquired from 350 to 2500 nm, Bilhalva et al. (2025b) obtained classification accuracies above 97% for parboiled rice Types 1–5 and Off‐Type using support vector machine and logistic regression algorithms. This previous investigation addressed a classification problem, in which samples were assigned to predefined commercial categories, rather than the quantitative prediction of individual physicochemical constituents.

More recently, Arf et al. (2026) evaluated broad‐range VIS–SWIR spectra from white, black, red, and parboiled rice using linear regression, random forest, gradient boosting, support vector machine, convolutional neural networks, and recurrent neural networks. The support vector machine model achieved an overall *R* ^2^ of 0.904, demonstrating the potential of machine‐learning algorithms for predicting rice physicochemical quality across different processing types. However, the study evaluated multiple rice categories jointly and emphasized comparisons among machine‐learning models and rice types rather than the individual predictability of each proximate‐composition attribute specifically within commercial parboiled rice samples.

Although these findings demonstrate the potential of spectroscopic methods, high performance reported for a specific dataset or attribute should not be interpreted as evidence of universal model accuracy or direct transferability. Predictive performance depends on the concentration range and distribution of the reference values, number and representativeness of samples, rice cultivar, processing conditions, spectral range, reference analytical method, preprocessing procedure, modeling algorithm, and validation strategy. Models developed under controlled conditions or using broad compositional ranges may achieve high coefficients of determination but may perform less accurately when applied to independent samples with narrower variability.

The prediction of constituents present at lower concentrations may also be more difficult because their spectral signals can be weak, indirect, or overlapped by the absorption bands of predominant components such as water and starch. In addition, grain size, surface characteristics, arrangement within the sample holder, light scattering, baseline variation, harvest season, processing conditions, and instrumental differences may affect spectral responses. Therefore, external validation using representative samples is essential for determining whether model performance is maintained beyond the calibration dataset.

The integration of VNIR–SWIR spectroscopy with chemometric analysis enables relevant information to be extracted from broad, highly correlated, and overlapping spectral bands. Partial least squares (PLS) regression is particularly suitable for modeling relationships between a large number of collinear spectral variables and physicochemical reference values. Spectral preprocessing may further reduce interferences associated with instrumental noise, light scattering, baseline shifts, grain arrangement, and sample heterogeneity. However, preprocessing does not necessarily improve all models, and its effectiveness must be evaluated separately for each target attribute (Lutz and Coradi 2022; H. P. Wang et al. 2022).

Previous spectroscopic investigations of rice have primarily focused on varietal or origin discrimination, physicochemical characterization, commercial‐type classification, individual‐grain visualization, or the prediction of specific processing attributes. In contrast, the present study focuses exclusively on parboiled rice representing different Brazilian commercial types and addresses a quantitative regression problem involving six proximate‐composition attributes. It systematically compares six spectral preprocessing strategies and evaluates PLS models through calibration, internal cross‐validation, and a held‐out test set.

Accordingly, this study aimed to evaluate the accuracy, predictive performance, and limitations of VNIR–SWIR spectroscopy combined with chemometric methods for the rapid and non‐destructive prediction of selected physicochemical attributes of parboiled rice representing different commercial types.

## 2 Materials and Methods

### 2.1 Obtaining the Samples

The experiment was conducted at the Agricultural Post‐Harvest Laboratory (LAPOS) of UFSM, Cachoeira do Sul campus, and at the Spectroscopy Laboratory of UFMS, Chapadão do Sul campus, using parboiled rice samples classified as Types 1–5 and Off‐Type. These classifications are defined according to Instruction No. 6 (Ministry of Agriculture, Livestock and Food Supply 2009) issued by the Ministry of Agriculture, Livestock and Food Supply (MAPA), which establishes the technical regulations and maximum permissible defect limits for processed parboiled rice in Brazil.

According to the Brazilian official rice classification standard, rice is categorized into groups according to its form of presentation, subgroups according to the processing method, classes according to grain dimensions, and commercial types according to quality and maximum defect tolerances (Ministry of Agriculture, Livestock and Food Supply 2009, 2012). Therefore, the terms “class” and “type” have distinct regulatory meanings. Grain classes, such as long‐fine, long, medium, short, and mixed, are determined by kernel dimensions, whereas Types 1–5 are determined by the maximum percentages of defects permitted for each rice subgroup.

Commercial type assignment was based on the percentage by mass of the physical defects, broken‐grain fractions, foreign matter, and impurities present in each sample, according to the maximum tolerance limits established for processed parboiled rice by Brazilian Instruction No. 6/2009. Type 1 represents the most restrictive defect limits, whereas progressively higher defect percentages are permitted from Types 2–5. Samples exceeding at least one of the maximum tolerance limits established for Type 5 were considered Off‐Type.

According to the maximum tolerance limits established for parboiled rice (Ministry of Agriculture, Livestock and Food Supply 2009), non‐gelatinized grains are considered one of the defects used for commercial type classification. However, because of the difficulty in obtaining samples containing this specific defect, non‐gelatinized grains were not included in the experimental samples.

The commercial type was assigned independently of the nutritional or proximate composition. Moisture, starch, protein, lipids, fiber, and ash were subsequently measured as physicochemical response variables and were not used to define the commercial type. Consequently, no individual nutrient was considered a dominant criterion for labeling the parboiled rice samples as Types 1 to 5.

For each of the six commercial categories—Types 1–5 and Off‐Type—a 2 kg sample was homogenized and divided into 100 subsamples of 20 g, resulting in a total of 600 subsamples. The measurements were performed on bulk samples of intact kernels, and the number of individual grains was not used as a standardization criterion.

Each commercial type was represented by one 2 kg bulk preparation, which was subsequently divided into 100 analytical subsamples. Therefore, the subsamples represented within‐batch analytical variation and should not be interpreted as independent production batches or biological replicates.

Cultivar identity was not included as an experimental factor or prediction target. Therefore, the predictive performance reported in this study applies to the evaluated sample population and should not be interpreted as evidence of model transferability across all rice cultivars. Validation using different cultivars, harvest seasons, production regions, and processing conditions is required before broader application.

### 2.2 Proximate Composition

The proximate composition, expressed as a percentage and including crude protein (PRO), moisture content (UMI), ether extract (LIP), crude fiber (FIB), ash (CIN), and starch (AMI), was determined in intact parboiled rice kernels from Type 1 to Type 5 and Off‐Type samples using a FOSS DS2500 NIR spectrometer (FOSS, Hillerød, Denmark). The analysis was performed in triplicate, as shown in Figure 1A. The samples were prepared and placed in the sampling capsule. The procedure involves illuminating the sample with radiation at a specific wavelength in the NIR range and measuring the energy differences emitted by the spectrometer and reflected from the sample to the detector.

**FIGURE 1:**
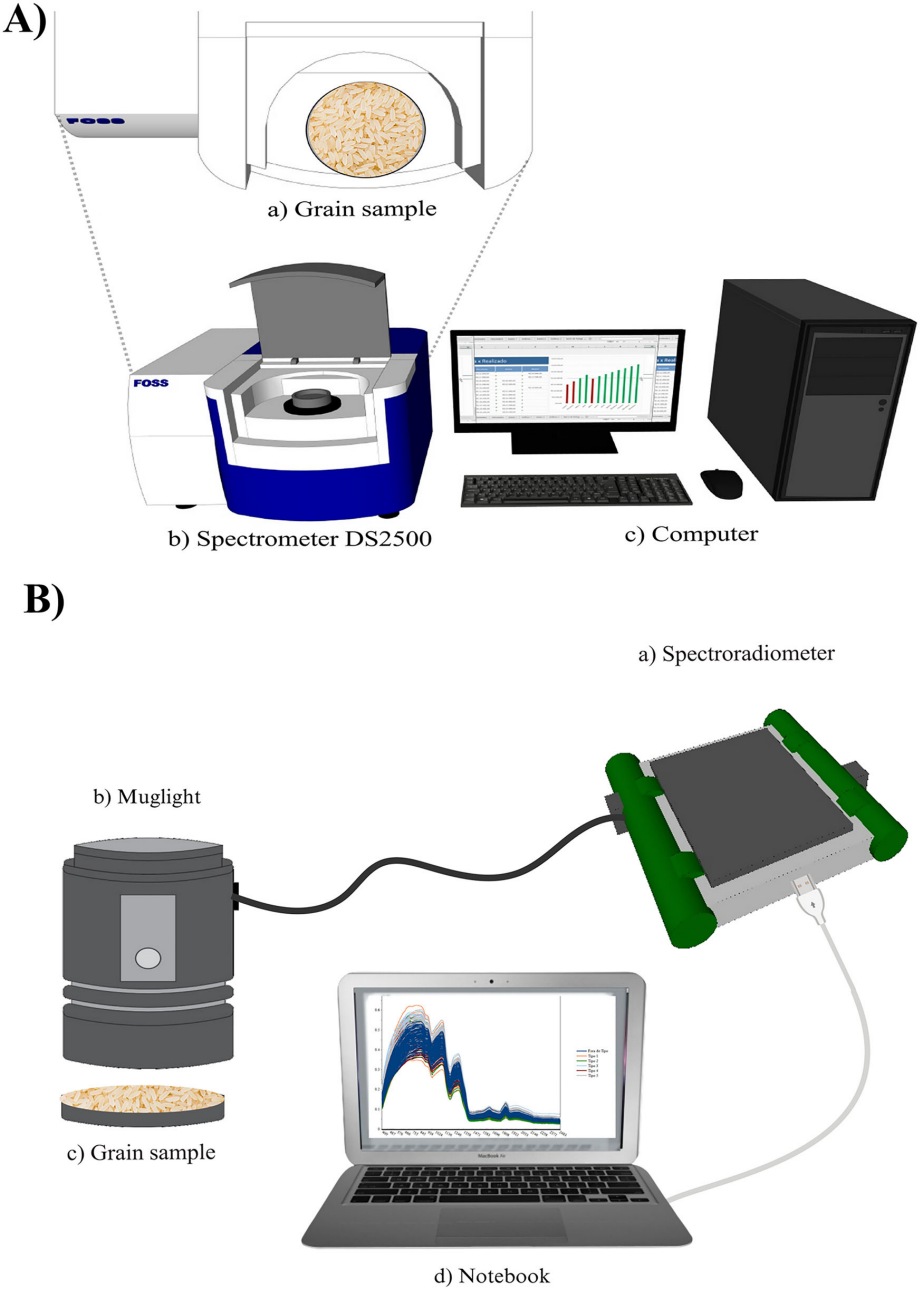
(A) Near‐infrared spectroscopy acquisition system for proximate composition and (B) Reflectance spectroscopy acquisition system.

The use of the FOSS DS2500 was consistent with the objective of evaluating a rapid and non‐destructive analytical workflow for industrial applications. The study did not aim to establish primary wet‐chemistry calibration equations. Instead, the measurements obtained using the standardized benchtop NIR system were used as comparative reference values to evaluate the ability of the FieldSpec VNIR–SWIR system to reproduce compositional information using a different non‐imaging spectral acquisition configuration.

Because the DS2500 estimates were not verified against primary wet‐chemistry methods in the present study, they were treated as comparative spectroscopic reference values rather than primary analytical reference values. Consequently, the reported PLS performance represents agreement between the FieldSpec measurements and the DS2500 estimates and should not be interpreted as absolute analytical accuracy relative to standardized chemical methods.

The spectral data were collected in reflectance mode across the spectral range of 400–2500 nm. After data collection, an analysis of variance (ANOVA) was conducted using the *F* test to assess significant differences between the treatments. Mean comparisons were made using the Scott–Knott test at a 5% probability level, utilizing Sisvar 5.8 software. The final averages are presented as boxplots.

### 2.3 Spectral Data

Reflectance spectra were acquired using a non‐imaging spectroradiometer (FieldSpec 4 Jr, Analytical Spectral Devices—ASD, Boulder, CO, USA) equipped with a Hi‐Brite Muglight accessory (Analytical Spectral Devices—ASD, Boulder, CO, USA). The Muglight contained a 4 W tungsten–quartz–halogen light source, with an approximate color temperature of 2900 K and a 12 mm illumination spot, and was designed to minimize interference from ambient light and specular reflection. The spectra were acquired over the wavelength range of 350–2500 nm. The spectral resolution was 3 nm from 350 to 700 and 30 nm from 1400 to 2100 nm, with sampling intervals of 1.4 nm from 350 to 1050 and 2 nm from 1051 to 2500 nm (da Silva et al. 2018).

From an operational perspective, the non‐imaging procedure used in this study required only the placement of a fixed 20 g subsample of intact rice kernels in a Petri dish, without grinding, extraction, chemical reagents, or sample destruction. These characteristics support its use for rapid bulk screening. However, because acquisition time, instrument costs, maintenance expenses, and consumable costs were not formally evaluated, the present study does not claim that this approach is universally faster, simpler, or less expensive than conventional NIR or Raman systems.

Before sample measurements, the instrument was calibrated using a white barium sulfate reference plate, which reflects approximately 100% of diffuse light. The reference spectrum was recorded and stored for the subsequent determination of sample reflectance. The white reference plate was used exclusively during instrument calibration and was removed before the rice samples were measured.

For each spectral measurement, a fixed 20 g subsample of intact parboiled rice kernels was placed in an 8 cm‐diameter Petri dish positioned directly in the Muglight sample holder. The Petri dish was not placed on the white reference plate. The same sample mass, Petri dish, and measurement position were maintained throughout the experiment. Three spectral readings were recorded for each subsample, and the mean spectrum was used for subsequent preprocessing and PLS modeling.

Spectral acquisition was controlled using RS^3^ Spectral Acquisition Software (Analytical Spectral Devices—ASD, Boulder, CO, USA), proprietary software supplied by the instrument manufacturer for operating the FieldSpec spectroradiometer and recording and storing the reflectance data. The spectral files were subsequently imported into ViewSpec Pro software and exported in .txt format for further preprocessing and statistical modeling. Details of the acquisition system and measurement procedure are presented in Figure 1B.

The total operational time per subsample, including weighing, sample positioning, replicate acquisition, reference calibration, and data export, was not formally recorded. Therefore, the present study does not provide a quantitative comparison of acquisition time or operating costs with conventional NIR, Raman spectroscopy, or hyperspectral imaging systems.

### 2.4 Spectral Preprocessing

Random noise, baseline shifts, and light‐scattering effects may result from instrumental conditions and physical differences among rice samples, including variations in grain size, surface characteristics, orientation, and packing within the sample holder. Spectral preprocessing may reduce these effects and improve model performance, although its effectiveness depends on the physicochemical attribute being predicted (L. Wang et al. 2024).

The preprocessing approaches were selected a priori according to the main sources of variation commonly observed in diffuse‐reflectance spectra. Baseline offset correction (BL) was evaluated to remove additive shifts in spectral intensity. Standard Normal Variate (SNV) and Multiplicative Scatter Correction (MSC) were selected to reduce multiplicative scattering and differences in spectral amplitude associated with sample heterogeneity and optical‐path variation. Savitzky–Golay smoothing (SSG) was used to reduce random variation while preserving the shape of spectral bands, whereas the first Savitzky–Golay derivative (1DSG) was evaluated to reduce baseline variation and enhance subtle or overlapping spectral features. Untreated spectra were retained as a reference to determine whether preprocessing improved model performance.

Six spectral treatments were systematically applied: (i) no preprocessing; (ii) BL; (iii) SNV; (iv) MSC; (v) SNV + BL + SSG, using a nine‐point window and a second‐order polynomial; and (vi) 1DSG, using an 11‐point window and a second‐order polynomial. Spectral preprocessing was performed using The Unscrambler X, version 10.4 (CAMO Software, Oslo, Norway).

SNV was applied by centering and scaling each individual spectrum according to its mean and standard deviation. MSC was performed by regressing each spectrum against the mean spectrum of the calibration set to correct additive and multiplicative scattering effects. Savitzky–Golay smoothing was based on local least‐squares polynomial fitting using a nine‐point window and a second‐order polynomial, whereas the first Savitzky–Golay derivative was calculated using an 11‐point window and a second‐order polynomial. For the combined treatment, the methods were applied sequentially as SNV, baseline offset correction, and Savitzky–Golay smoothing. All preprocessing procedures were performed using the standard, unmodified algorithms available in The Unscrambler X.

Model development and preprocessing comparisons were based on calibration and internal cross‐validation performance, considering *R* ^2^, correlation coefficients, RMSEC, and RMSECV. RMSEP was calculated only during the subsequent held‐out test‐set evaluation and was not used for model fitting.

### 2.5 PLS Regression

PLS regression is a multivariate calibration technique widely used in studies focused on modeling the relationships between observed variables (x, spectrum) and their corresponding physicochemical compositions (y). PLS can project the observed data onto a small number of latent variables, which cannot be directly measured or observed. Thus, PLS combines regression, dimensionality reduction, and modeling (Lou et al. 2024).

By maximizing the covariance between X and Y, the PLS algorithm switches between regression and compression steps to generate a set of orthogonal factors, referred to as optimal factors. These factors are evaluated using a calibration set (Guo et al. 2019; Hao et al. 2019). The PLS model decomposes the spectral and response matrices into latent‐variable scores and loadings and establishes an inner regression relationship between their score matrices, as follows:

$$X=TP^{T}+E$$

(1) $$Y=UQ^{T}+F$$

U=TB+H

Where X(*n* × *m*) is the independent variable (spectral matrix); U(*n* × *k*) is the score matrix describing the dependent variable Y; T(*n* × *k*) is the score matrix describing the dependent variable X; P(*m* × *k*) is the eigenvector matrix of the independent variable; Q(*p* × *k*) is the eigenvector matrix of the dependent variable; E(*n* × *m*), F(*n* × *p*), and H(*n* × *k*) are the residual matrices; and B(*k* × *k*) is the PLS regression coefficient. The PLS models were developed using The Unscrambler X, version 10.4 (CAMO Software, Oslo, Norway).

The dataset was partitioned into a calibration set and a held‐out test set using the Kennard–Stone algorithm. Seventy‐five percent of the samples were assigned to calibration and internal cross‐validation, while the remaining 25% were reserved exclusively for final test‐set evaluation.

Although 600 subsamples were initially acquired, 106 samples were excluded as outliers during the original model‐development procedure. Although 600 subsamples were initially acquired, 106 observations were excluded as outliers during the original data‐analysis workflow, resulting in a final dataset of 494 observations. The available records did not retain the diagnostic statistic, threshold, or complete sequence used for this exclusion. Therefore, the reproducibility of the original outlier‐screening procedure is limited and is explicitly acknowledged as a methodological limitation. The final modeling dataset therefore comprised 494 samples. The Kennard–Stone algorithm assigned 370 samples to the calibration set and internal cross‐validation and 124 samples to the held‐out test set. Samples assigned to the held‐out test set were not used during model development or preprocessing selection.

The exact diagnostic statistic and threshold originally used for outlier identification were not retained and could not be recovered; therefore, this aspect is acknowledged as a limitation of the study.

Samples in the held‐out test set were not used during model development or preprocessing selection. Because both subsets originated from the same sample population, the test‐set evaluation should not be interpreted as fully external validation based on independent production batches.

$$X=TP^{2}+E\;Y=UQ^{2}+F\;U=TB+H$$

where *X*(*n* × *m*) represents the independent variable (spectral matrix), **U**(*n* × *k*) is the score matrix for the dependent variable *Y*, and *T*(*n* × *k*) is the score matrix for the independent variable XP(*m* × *k*), and Q(*p* × *k*) are the *X*‐ and *Y*‐loading matrices, respectively. *E*(*n* × *m*), *F*(*n* × *p*), and *H*(*n* × *k*) are the residual matrices, and *B*(*k* × *k*) is the regression coefficient matrix of the PLS model (Lee et al. 2022).

### 2.6 Software Used

To ensure accurate data analysis and visualization, the following software tools were used throughout the study: (i) The Unscrambler X (version 10.4, CAMO Software, Oslo, Norway): Used for spectral data preprocessing with techniques such as SNV, MSC, BL, Savitzky‐Golay smoothing, and first derivative (1DSG). Also employed to develop and validate PLS regression models, including calibration and internal cross‐validation; (ii) RS^3^ (Analytical Spectral Devices—ASD): proprietary software used to control the FieldSpec 4 Jr spectroradiometer during non‐imaging VNIR–SWIR spectral acquisition and to record the reflectance spectra; (iii) ViewSpec Pro: utilized to extract and convert spectral data from the RS^3^ proprietary format into .txt files for further preprocessing and statistical modeling.

(iv) Applied to conduct ANOVA using the *F*‐test and to perform mean comparisons via the Scott–Knott test (*p* < 0.05) on proximate composition data; (v) OriginPro 2022 (OriginLab Corporation, Northampton, MA, USA): Used for the generation of all graphical outputs, including boxplots of proximate composition, spectral curves, and predicted versus reference scatterplots. It ensured high‐quality visualizations with statistical annotations; (vi) Microsoft Excel 2019 (Microsoft Corp., Redmond, WA, USA): Used for data organization, initial cleaning of spectral and proximate‐composition datasets, and basic descriptive statistical calculations prior to modeling.

## 3 Results and Discussion

### 3.1 Proximate Composition of Parboiled Rice Grains

Samples of parboiled rice from Type 1 to Type 5 and Off‐Type showed significant differences (*p* ≤ 0.05) in moisture, starch, protein, lipids, fiber, and ash content, as shown in Figure 2. Moisture content ranged between 11.19% (Type 1) and 12.65% (Type 5), with an average of 11.94%, which is below the maximum threshold of 14%, considered ideal for maintaining rice quality during storage and processing. Moisture levels exceeding 14% can increase the likelihood of breakage and compromise the quality of the final product. This moisture range is essential for maintaining the culinary and milling properties of parboiled rice, as well as ensuring an optimal shelf life (Moraes et al. 2023; Muchlisyiyah et al. 2023). These findings are consistent with studies suggesting that moisture levels for parboiled rice should be maintained between 12% and 14% to optimize milling performance and minimize grain breakage during processing (Alim et al. 2024).

**FIGURE 2:**
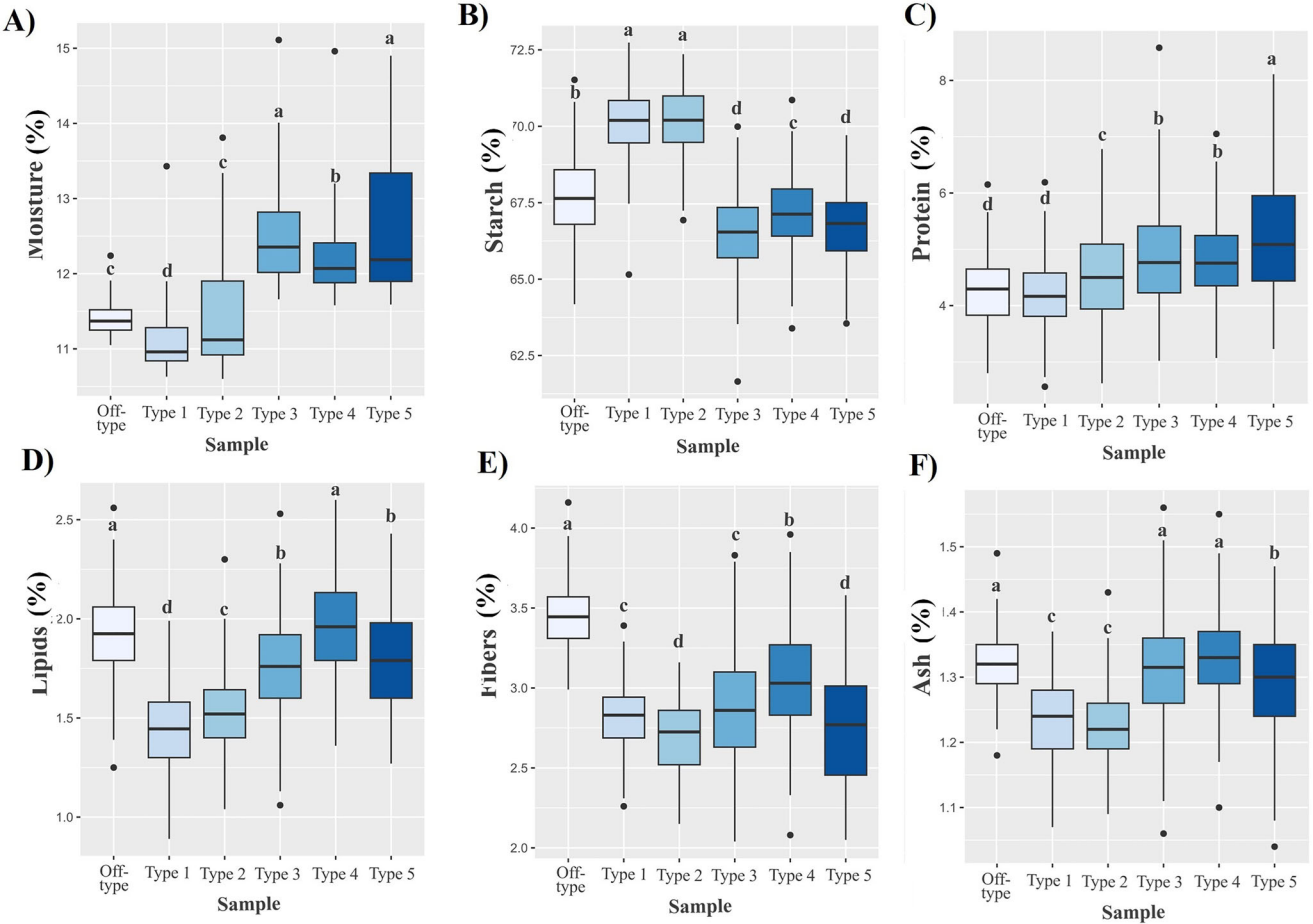
Boxplot for the averages of parboiled rice (Type 1 to Type 5 and Off‐Type) according to the contents of (A) Moisture; (B) Starch; (C) Protein; (D) Lipids; (E) Fibers; (F) Ash. Means followed by the same letter do not differ from each other at the 5% probability level.

Starch, the primary component of rice grains, consists of two main glucose polymers: amylopectin and amylose, which are crucial for both the technological quality and consumption of rice (Ansah et al. 2023). The highest starch content was found in samples from Type 1 (70.11%) and Type 2 (70.16%), while the lowest content was observed in the Type 3 sample (66.52%), followed by the Type 5 sample (66.73%). The lower starch contents observed in Types 3 and 5 indicate compositional variation among the commercial types. However, fungal incidence, insect damage, and mycotoxin concentrations were not measured in the present study. Therefore, the causes of these differences cannot be established from the current dataset and may reflect combined effects of raw‐material variability, processing conditions, and the physical‐defect composition of each sample.

The highest protein concentration was observed in samples from Type 5 (5.25%), while the lowest was in samples from Type 1 (4.19%) and Off‐Type (4.26%).

Figures 3, 4, 5 display the spectra of unprocessed data for moisture, starch, protein, lipids, fiber, and ash from 600 parboiled rice samples, grouped according to commercial type (Type 1, Type 2, Type 3, Type 4, Type 5, and Off‐Type). Each graph (1–6) shows spectral curves that vary according to the wavelength (350–2500 nm) on the *x*‐axis and reflectance (0–0.6) on the *y*‐axis, with colors representing the concentration of each component (%) according to the color legend. These graphs are effective visual tools that help in understanding how the proximate composition of parboiled rice is reflected in the spectra. This representation is useful for analyzing variability, identifying patterns, and comparing different types of rice.

**FIGURE 3:**
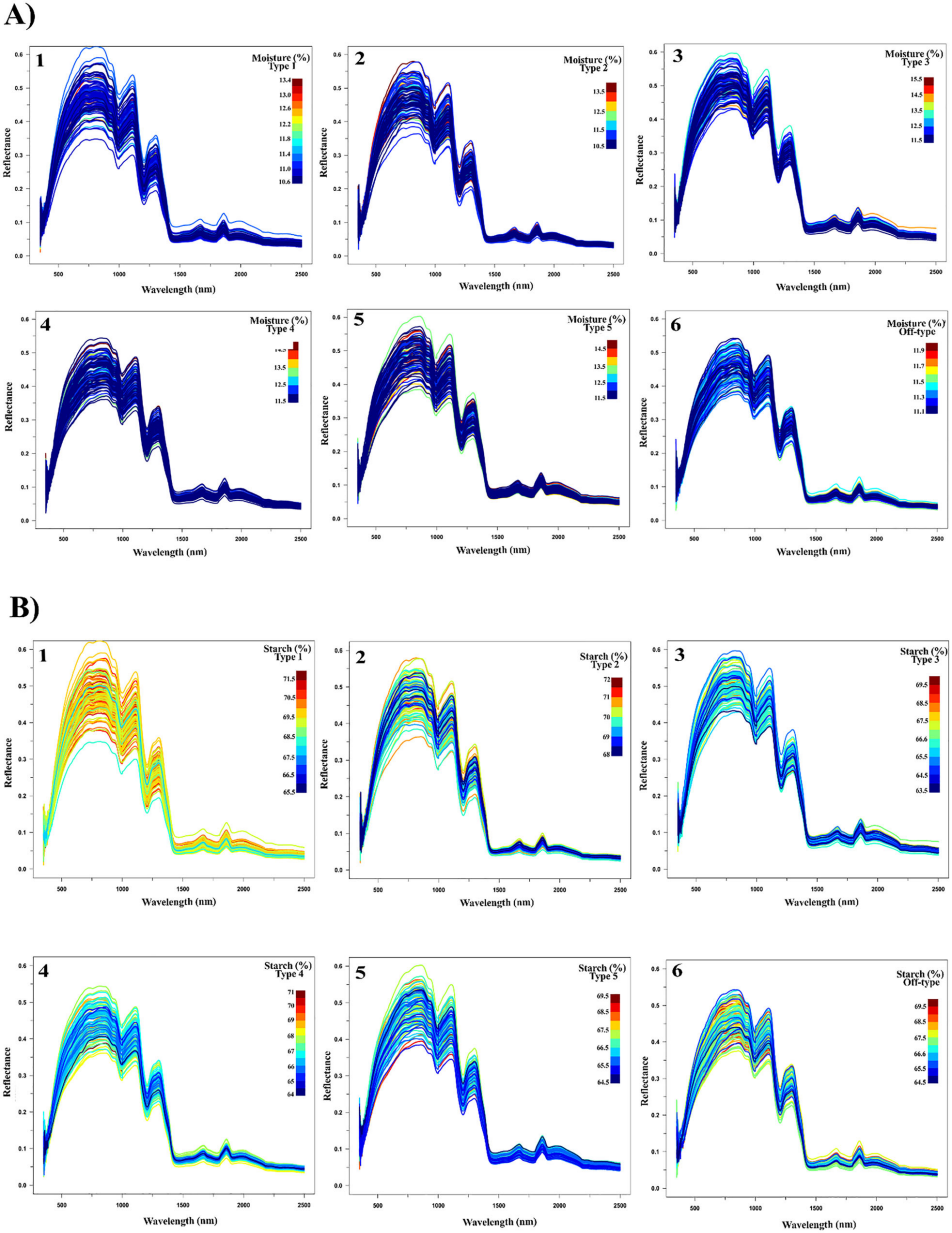
(A) Spectra of data without preprocessing of moisture from rice samples of different types: (A) Type 1, (B) Type 2, (C) Type 3, (D) Type 4, (E) Type 5, and (F) Off‐Type, and (B) spectra of data without preprocessing of starch from rice samples of different types: (A) Type 1, (B) Type 2, (C) Type 3, (D) Type 4, (E) Type 5, and (F) Off‐Type.

**FIGURE 4:**
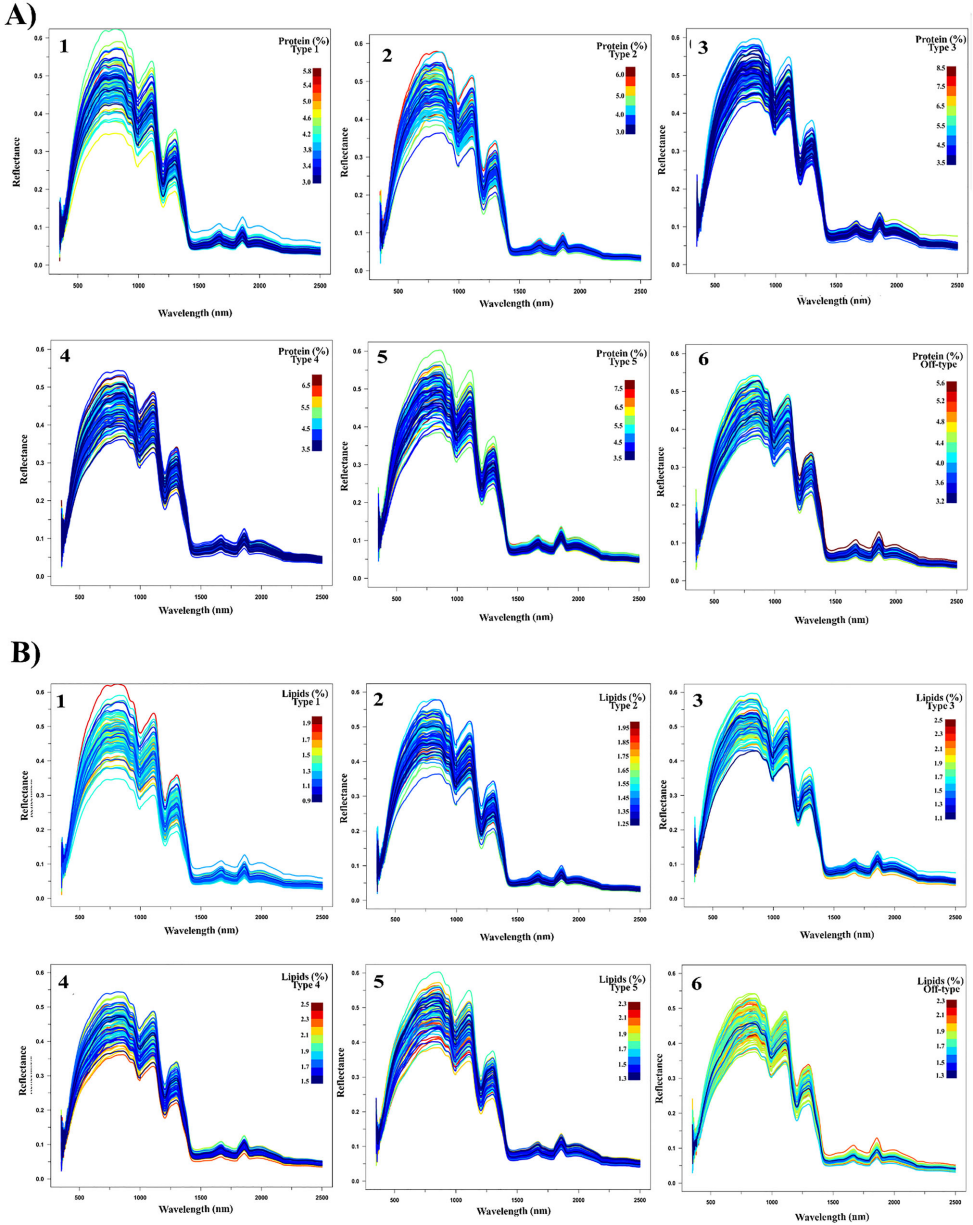
(A) Spectra of data without preprocessing of protein from rice samples of different types: (A.1) Type 1, (A.2) Type 2, (A.3) Type 3, (A.4) Type 4, (A.5) Type 5, and (A.6) Off‐Type, and (B) spectra of data without preprocessing of lipids from rice samples of different types: (B.1) Type 1, (B.2) Type 2, (B.3) Type 3, (B.4) Type 4, (B.5) Type 5, and (B.6) Off‐Type.

**FIGURE 5:**
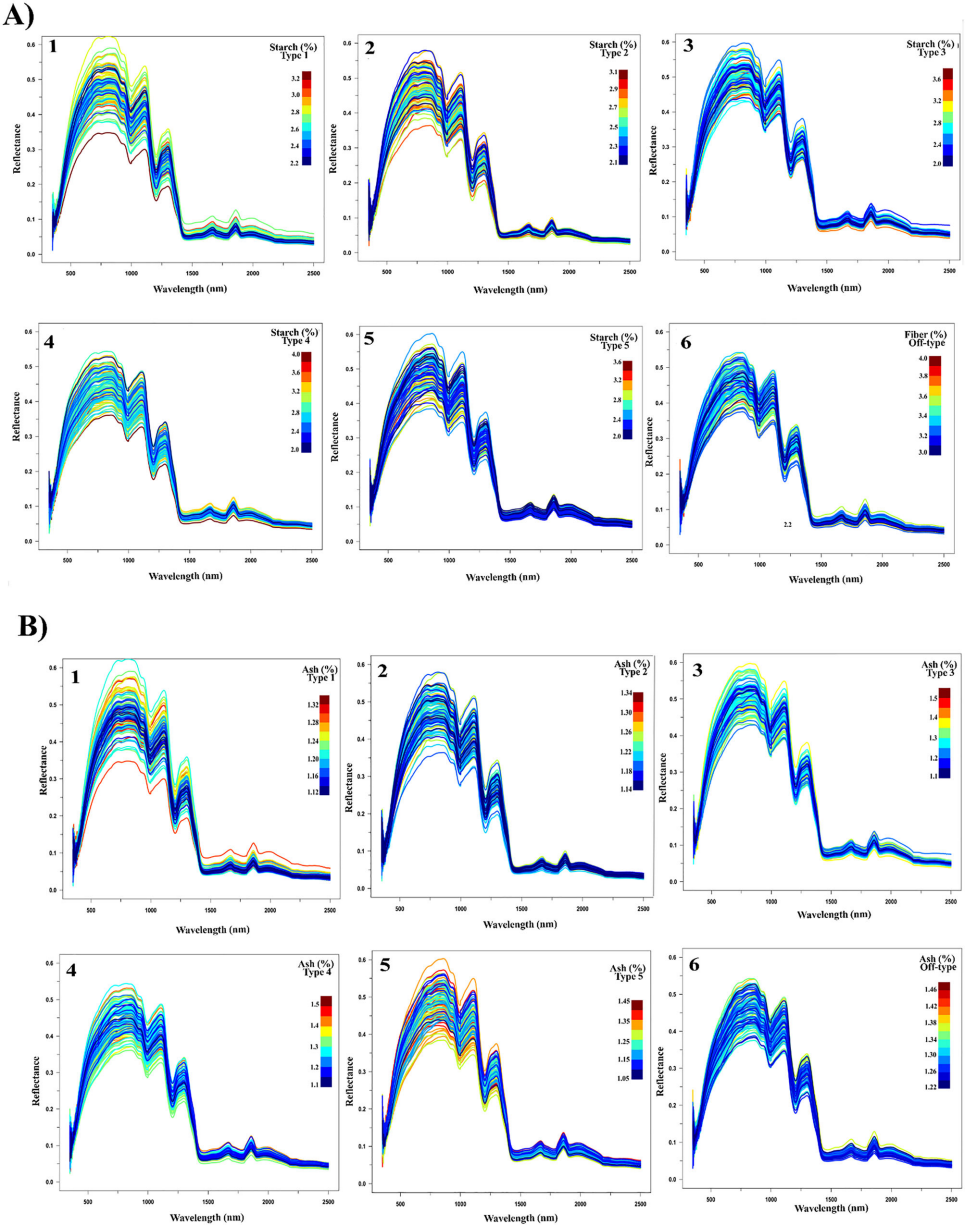
(A) Spectra of data without preprocessing of fibers from rice samples of different types: (A.1) Type 1, (A.2) Type 2, (A.3) Type 3, (A.4) Type 4, (A.5) Type 5, and (A.6) Off‐Type, and (B) spectra of data without preprocessing of ash from rice samples of different types: (B.1) Type 1, (B.2) Type 2, (B.3) Type 3, (B.4) Type 4, (B.5) Type 5, and (B.6) Off‐Type.

The higher protein concentration observed in Type 5 should not be interpreted as evidence of superior commercial quality, because the commercial type is determined by physical defects rather than nutrient content. The causes of the observed protein differences were not directly investigated and may reflect variability in the raw material, cultivar composition, and processing history.

The spectral profiles showed visual differences in reflectance intensity and dispersion among the commercial types, particularly near water‐related absorption bands at approximately 1450 and 1950 nm. However, these observations are descriptive because no wavelength‐by‐wavelength statistical test was performed. Therefore, they should not be interpreted as independent spectral validation of the Brazilian commercial classification system.

The intensity of the color indicates the concentration of the component across the spectral range. More intense colors represent higher concentrations of the component. Each component interacts differently with light at various wavelengths, meaning some wavelengths are more sensitive to the presence of specific components than others. For instance, water strongly absorbs light at specific wavelengths in the NIR region, while other components may have absorption peaks in other regions of the spectrum. Visual differences were observed among the spectral profiles of the commercial types, particularly in reflectance intensity and spectral dispersion. However, these visual observations should not be interpreted as statistically significant spectral differences because no wavelength‐by‐wavelength significance test was performed.

It is important to note that non‐gelatinized grains are included among the defects considered in the Brazilian classification standard for parboiled rice. However, this specific defect was not represented in the experimental samples because of the difficulty in obtaining sufficient quantities of affected grains. This limitation does not invalidate the commercial classification adopted in the study, because rice samples may be assigned to lower commercial types or classified as Off‐Type when the tolerance limit for any other regulated defect is exceeded. Therefore, the classifications used in this study were based on the other physical defects present in the samples, including broken, damaged, burned, blackened, streaked, spotted, stained, or non‐parboiled grains, as applicable.

### 3.2 Interpretation of Spectra for the Quality of Parboiled Rice Grains

Figure SA, panel B, presents the parboiled rice spectra after baseline offset correction. Comparison of panels A and B indicated visually reduced spectral dispersion and more uniform baseline alignment after preprocessing. However, no quantitative signal‐to‐noise ratio was calculated. The other treatments—SNV, MSC, SNV + BL + SSG, and 1DSG—are shown in panels C–F.

The combined SNV + BL + SSG treatment (Figure SA, panel E) visually reduced spectral dispersion and overlap. The first derivative treatment (panel F) enhanced subtle spectral variations and reduced baseline effects; however, derivative transformations may also amplify random noise when the analytical signal is weak. Negative derivative values represent rates of spectral change rather than physical reflectance values and should therefore be interpreted as transformed spectral features.

Figure 6 shows the means of the original spectra corresponding to different types of parboiled rice (Types 1–5 and Off‐Type) recorded in the range from 750 to 2500 nm. The shape of the diffuse reflectance spectrum for the rice sample results from changes in energy vibrations in the form of overtones, bends, and stretches (Hayati et al. 2021). In other words, the spectral shape is a direct consequence of the molecular vibrations of its components, which absorb and reflect light in a specific manner. It is worth noting that the band shapes were similar across treatments, and the main visual differences in the parboiled rice spectra were observed in reflectance intensity. The reference for interpreting the spectral curves is shown in Table SB.

**FIGURE 6:**
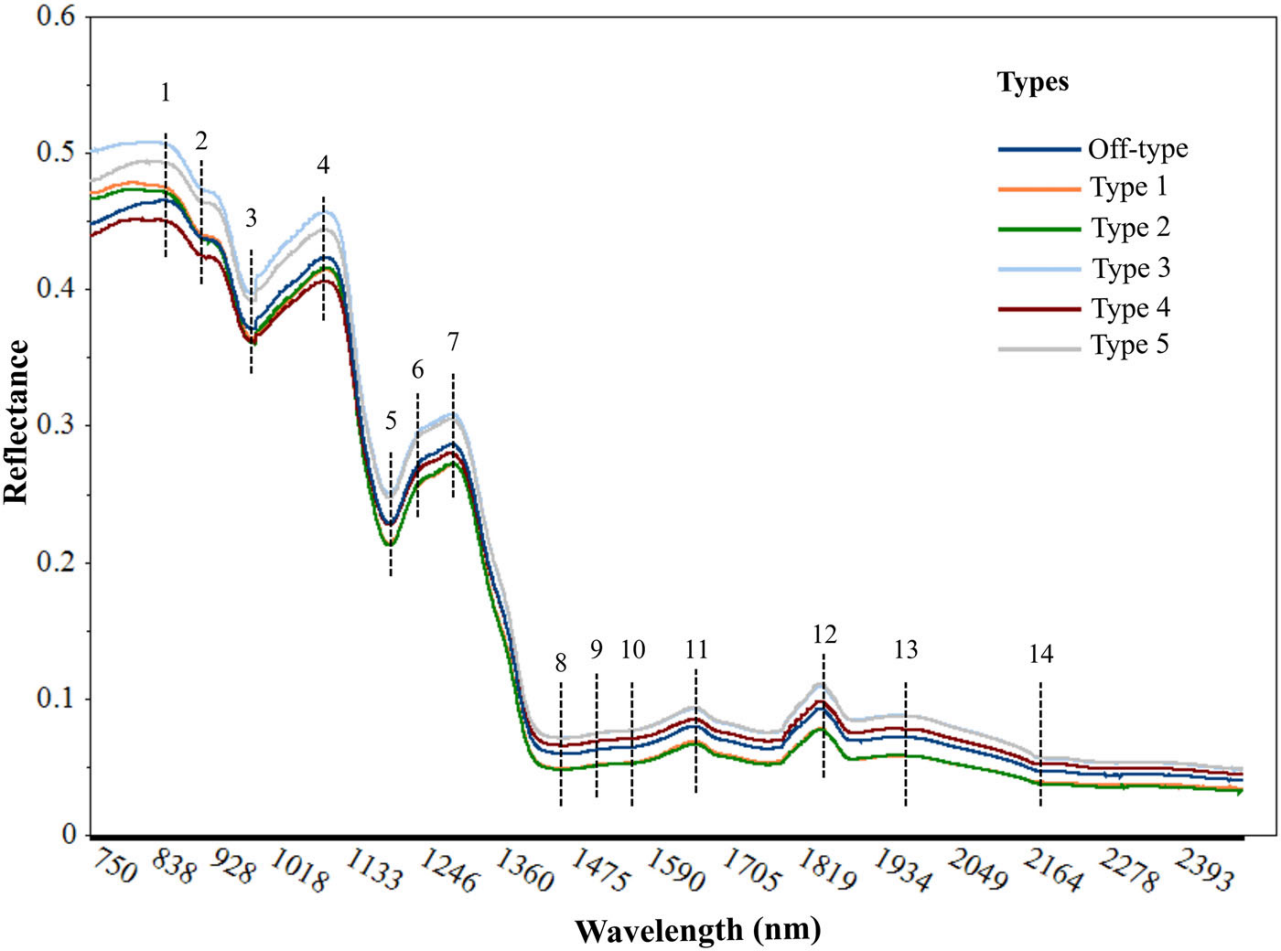
Average of the original spectra corresponding to the different types of parboiled rice studied (Type 1 to Type 5 and Off‐Type). The numbers are the spectral absorption bands associated with components present in rice grains (Table S2).

The wavelength range used (750–2500 nm) encompasses chemical bonds such as C‐H, N‐H, and O‐H, which can be associated with starch polysaccharides. When analyzed with chemometrics, these data can support qualitative and quantitative chemometric analyses (Liu et al. 2020). According to Liu et al. (2020) and Teye and Amuah (2022), the range between 850 and 880 nm represents the third overtone of N‐H (RNHR) and C‐H, which is associated with the protein and amylose content in rice samples. In the graph, the wavelength found was between 835 and 900 nm, with an average wavelength of 867.5 nm, aligning well with the findings of the authors; thus, this range can be characterized as indicative of protein and amylose content in rice grains. Amylose is a polysaccharide that, along with amylopectin, constitutes starch (Lima et al. 2021; Cui et al. 2022). Teye and Amuah (2022), when detecting integrity fraud and adulteration of rice varieties through chemometric analysis of pocket NIR spectra, determined that wavelengths around 900–950 nm in rice grains suggest the presence of water. However, the presence of the aleurone layer may influence spectral information collection, complicating accurate detection.

The absorption peak at 985 nm in the NIR spectrum is related to the stretching vibration of the NH bond in proteins (Wadood et al. 2019). Likewise, the peak at 1200 nm is attributed to the stretching vibration of CH in lipids, starch, and proteins (Wadood et al. 2019; Wei et al. 2021). Additionally, absorption peaks at 1460 and 1940 nm correspond to the bending vibration of the OH bond in water (Rachmawati and Rafi 2017; Hayati et al. 2021). The first harmonic of the O─H molecule is clearly visible in this wavelength range; thus, it can be asserted that the water absorption band occurs in the wavelength range from 1400 to 1900 nm. This finding is also supported by some literature stating that water absorption occurs at wavelengths from 1420 to 1490 nm and 1900 to 1980 nm. Spectral bands within the 2200–2300 nm region have been associated with combination and overtone vibrations involving C–H and O–H bonds in carbohydrate‐related compounds (Yang et al. 2018; Awanthi et al. 2019; Islam et al. 2021).

The C─H─O composition is linked to the nutritional content of carbohydrates in rice. Additionally, the N─H molecular structure vibrates (first overtone) within the wavelength range of 1500–1600 nm, while the molecular structure of organic acids vibrates (first harmonic and bending) in the wavelength ranges of 1400, 1800, and 2100 nm (Hayati et al. 2021).

These spectral variations support the investigation of relationships between VNIR–SWIR reflectance and the physicochemical composition of parboiled rice across different commercial types. However, the present study focused on quantitative compositional prediction rather than on the classification of commercial types.

The prominent water‐related spectral regions observed in the present study are consistent with the findings of Song et al. (2023), who obtained high predictive performance for rice moisture using hyperspectral imaging in the 900–1700 nm range. However, their spatially resolved imaging configuration and variable‐selection procedure differ from the non‐imaging full‐spectrum approach evaluated here.

### 3.3 Calibration Models and Internal Cross‐Validation

The ranges of proximate composition values (minimum and maximum) for each physicochemical variable—moisture, starch, protein, lipids, fiber, and ash—used in the calibration and validation sets are presented in Table SA. These ranges reflect the natural variability of the dataset and confirm that the predictive models were developed and tested across representative compositional intervals. As the dataset was partitioned using the Kennard–Stone algorithm implemented in The Unscrambler, the specific descriptive statistics (e.g., mean and standard deviation) for each subset were not externally extracted. However, the inclusion of the observed min–max ranges helps ensure transparency regarding data coverage and model generalizability.

For the quantitative determination of the components of the proximate composition, mathematical models were created using the PLS regression algorithm. According to Buvé et al. (2022), PLS is widely used in the multivariate study of various products, making it possible to assess the quality patterns of food.

Six PLS models, corresponding to the six spectral preprocessing conditions, were developed for each physicochemical constituent and evaluated using *R* ^2^, correlation coefficients, RMSEC, and RMSECV.

Regarding moisture, MSC and untreated spectra provided the most favorable results during calibration and internal cross‐validation. During calibration, *R* ^2^ values were 0.562 and 0.560, correlation coefficients were 0.750 and 0.748, and RMSEC values were 0.389 and 0.390 for MSC and untreated spectra, respectively. During internal cross‐validation, *R* ^2^ values were 0.539 and 0.535, correlation coefficients were 0.733 and 0.732, and RMSECV values were 0.400 and 0.401, respectively. Thus, MSC showed slightly better performance, although untreated spectra produced comparable results.

For starch, the most favorable results were obtained using untreated spectra and SNV. During calibration, both approaches resulted in *R* ^2^ values above 0.680 and correlation coefficients above 0.824, with RMSEC values of 0.976 for untreated spectra and 0.980 for SNV. During internal cross‐validation, *R* ^2^ values remained above 0.655 and correlation coefficients above 0.808, with RMSECV values of 1.016 and 1.020, respectively. These results indicate that SNV and untreated spectra preserved relevant information for starch prediction.

For the protein and lipid components, both SNV and MSC methods demonstrated similar performances, establishing them as the best options. However, protein was the component that showed the poorest fit to the models, with *R* ^2^ consistently below 0.166, suggesting limitations in the employed methods for accurately predicting this component. In contrast, for lipids, the SNV method slightly outperformed MSC, with an *R* ^2^ of 0.488 compared to 0.481 for MSC, indicating a marginally better fit for this component.

For fiber, MSC provided the best calibration and internal cross‐validation performance. The calibration model showed an *R* ^2^ of 0.360, a correlation coefficient of 0.600, and an RMSEC of 0.259. During internal cross‐validation, the corresponding values were *R* ^2^ = 0.333, correlation = 0.574, and RMSECV = 0.266. These results indicate limited quantitative predictability despite some improvement after scatter correction.

For ash, MSC and untreated spectra showed similar performance. The calibration results reached an *R* ^2^ of approximately 0.450, a correlation coefficient of 0.670, and an RMSEC of 0.049, while internal cross‐validation resulted in an *R* ^2^ of approximately 0.425, a correlation coefficient of 0.650, and an RMSECV of 0.051. Because minerals do not exhibit strong direct absorption in the VNIR–SWIR region, ash prediction was likely based on indirect correlations with organic constituents and physical sample characteristics.

The effect of spectral preprocessing depended on the chemical concentration, spectral intensity, and physical variability associated with each physicochemical attribute. MSC and SNV reduce multiplicative scattering and differences in spectral amplitude caused by variations in grain packing, orientation, surface properties, and sample heterogeneity. Consequently, these methods were beneficial for attributes whose spectral responses were influenced by both chemical absorption and physical scattering effects, particularly moisture and lipids.

For starch, the original spectra and SNV showed the best overall performance. Starch is the major constituent of rice and presents broad spectral features associated mainly with C–H and O–H molecular vibrations. Therefore, its spectral information was comparatively strong even without preprocessing. More complex combinations of preprocessing did not consistently improve starch prediction and may have reduced useful differences in reflectance intensity.

In contrast, protein showed weak performance with all preprocessing methods. Protein was present at relatively low concentrations and within a narrow compositional range, while its N–H absorption bands overlapped with signals from water, starch, and other organic constituents. Thus, scatter correction and baseline adjustment could reduce physical spectral interference but could not create sufficient chemical contrast for accurate protein prediction.

The limited performance observed for fiber may also be related to its low concentration and overlapping C–H and O–H absorption bands. MSC provided some improvement by correcting scattering associated with differences in grain structure and sample arrangement, but the remaining spectral overlap restricted quantitative performance. Ash prediction was also limited because minerals do not exhibit strong direct absorption bands in the VNIR–SWIR region. Therefore, ash was predicted indirectly through correlations with organic constituents and physical sample characteristics.

Savitzky–Golay smoothing and derivative preprocessing reduced baseline variation and enhanced subtle spectral features. However, derivative treatments may also amplify spectral noise when the analytical signal is weak, which helps explain why these methods did not consistently improve all physicochemical models. Overall, the results demonstrate that the most appropriate preprocessing method depends on the balance between physical scattering correction, preservation of useful spectral intensity, and the strength of the chemical information associated with each constituent.

Model performance was interpreted through the combined assessment of *R* ^2^, correlation coefficients, RMSEC, and RMSECV rather than through a single universal threshold. Similar RMSEC and RMSECV values generally indicate comparable error levels between calibration and internal cross‐validation, whereas a substantial increase in validation error relative to calibration error would provide stronger evidence of overfitting.

The results indicate that the usefulness of preprocessing depended on the target constituent. MSC and SNV were advantageous when scattering and differences in spectral amplitude contributed substantially to sample variation. In contrast, raw spectra or baseline correction preserved useful information for constituents with stronger spectral signals, particularly starch. Nevertheless, the low coefficients of determination observed for protein and fiber demonstrate that preprocessing alone could not compensate for narrow compositional ranges and overlapping absorption bands.

The limited protein performance observed in the present study contrasts with Hayati et al. (2021), who reported a correlation coefficient of 0.90 and an RPD of 3.19 for rice protein prediction using NIR–PLS. This difference may be associated with differences in the sample population, compositional range, spectral configuration, and reference method. Similarly, the high prediction coefficients reported by Taghinezhad et al. (2025) for starch gelatinization and head rice yield were obtained for specific processing attributes under controlled parboiling conditions, emphasizing the dependence of model performance on the target variable and experimental design.

### 3.4 Held‐Out Test‐Set Evaluation

The original dataset was partitioned using the Kennard–Stone algorithm. Seventy‐five percent of the samples were assigned to calibration and internal cross‐validation, while the remaining 25% were reserved as a held‐out test set and were not used during model development. Because the calibration and test subsets originated from the same experimental sample population, this evaluation does not represent fully external validation using independent production batches, harvest periods, or geographical origins.

Predictive performance differed substantially among the physicochemical attributes, as shown in Table SD and Figures SH–SM. For moisture, MSC, BL, and untreated spectra showed similar test‐set performance, with *R* ^2^ values of approximately 0.627 and RMSEP values below 0.37 percentage points. For starch, BL produced the best test‐set result, with *R* ^2^ = 0.740 and RMSEP = 0.902 percentage points.

Protein showed the lowest predictability, reaching a maximum test‐set *R* ^2^ of 0.112 with SNV + BL + SSG. For lipids, SNV and MSC produced the most favorable results. Fiber also showed limited predictability; SNV + BL + SSG produced the highest *R* ^2^ value of 0.248, whereas MSC produced a slightly lower RMSEP. For ash, SNV + BL + SSG resulted in the best test‐set performance, with *R* ^2^ = 0.492 and RMSEP = 0.045 percentage points.

Song et al. (2023) reported higher coefficients of determination for rice moisture using hyperspectral imaging, whereas Arf et al. (2026) obtained an overall *R* ^2^ of 0.904 using support vector machines across multiple rice categories. These results are not directly comparable with those of the present study because the investigations differed in sample variability, target attributes, spectral technology, and modeling algorithms. Nevertheless, they indicate that broader calibration populations and nonlinear approaches may improve performance when sufficient representative data are available.

The comparatively better performance of SNV and MSC for some attributes can be explained by their ability to reduce multiplicative scattering and spectral‐amplitude variation caused by grain orientation, surface characteristics, packing, and sample heterogeneity. However, these transformations did not consistently improve all attributes because model performance also depended on constituent concentration, spectral‐band overlap, and the range of reference values.

Overall, starch and moisture showed the greatest potential for rapid screening, whereas protein and fiber exhibited limited quantitative predictability. Lipid and ash models showed moderate performance but require further improvement. The results should therefore be interpreted as an assessment of model performance within the studied sample population. Validation using independent industrial batches, broader compositional ranges, different cultivars, and primary chemical reference analyses is required before routine implementation.

## 4 Conclusions

This study supports the feasibility of combining non‐imaging VNIR–SWIR reflectance spectroscopy with PLS modeling for the preliminary, non‐destructive screening of selected physicochemical attributes of parboiled rice. Starch showed the highest predictive performance, particularly after baseline offset correction, while moisture also presented promising results for screening applications. Lipid and ash models showed moderate performance, whereas protein and fiber exhibited limited quantitative predictability. The effectiveness of spectral preprocessing depended on the target constituent: SNV and MSC reduced scattering effects in some models, while baseline correction or untreated spectra preserved relevant information for starch and moisture. The main limitations included narrow compositional ranges, overlapping spectral absorption bands, sample heterogeneity, the use of indirect NIR measurements as comparative reference values, the use of analytical subsamples originating from the same experimental population rather than independent production batches, and the inability to recover the exact diagnostic statistic, threshold, and complete analytical sequence used for outlier exclusion during the original model‐development workflow. Cultivar, harvest season, production region, and processing conditions were not evaluated as independent factors. Therefore, the proposed approach should be regarded as a preliminary screening method rather than a replacement for standardized quantitative analyses or a fully validated industrial system. Future studies should use primary wet‐chemistry reference methods, broader and more diverse calibration populations, independent production batches, fully documented outlier‐identification procedures, calibration‐transfer assessment, and comparisons with nonlinear machine‐learning algorithms.

## Author Contributions

**Nairiane dos Santos Bilhalva**: conceptualization, investigation, writing – original draft, methodology, validation, visualization, writing – review and editing, formal analysis, data curation. **Marisa Menezes Leal**: investigation, visualization, software, formal analysis, data curation, methodology. **Rosana Santos de Moraes**: investigation, formal analysis, data curation. **Dthenifer Cordeiro Santana**: validation, data curation, software, formal analysis. **Larissa Pereira Ribeiro Teodoro**: data curation, formal analysis, validation, methodology. **Paulo Eduardo Teodoro**: investigation, methodology, formal analysis, data curation. **Paulo Carteri Coradi**: conceptualization, investigation, funding acquisition, writing – original draft, methodology, validation, writing – review and editing, visualization, project administration, formal analysis, software, data curation, supervision, resources.

## Funding

The authors thank CAPES (Coordination for the Improvement of Higher Education Personnel)‐Financial Code 001, CNPq (National Council for Scientific Technological Development) (number 304966/2023‐1), and FAPERGS‐RS (Research Support Foundation of the State of Rio Grande do Sul) (number 24/2551‐0001150‐1) for funding the research projects and laboratories for carrying out the experiments.

## Conflicts of Interest

The authors declare no conflicts of interest.

## Supporting information




**Figure S1**: A) Original spectra in the near‐infrared region (350 ‐ 2500 nm) of parboiled rice samples (Type 1 to Type 5 and Off‐Type) without the use of pretreatments, B) Baseline offset, C) SNV, D) MSC, E) SNV+BL+SSG and F) 1DSG.

**Figure S2**: Reference values ​​(Yref) versus predicted values ​​(Ypred) from the model calibration set without preprocessing for 1) Moisture; 2) Starch; 3) Protein; 4) Lipids; 5) Fiber and 6) Ash.

**Figure S3**: Reference values ​​(Yref) versus predicted values ​​(Ypred) from the model calibration set preprocessed with BL for 1) Moisture; 2) Starch; 3) Protein; 4) Lipids; 5) Fiber and 6) Ash.

**Figure S4**: Reference values ​​(Yref) versus predicted values ​​(Ypred) from the model calibration set preprocessed with SNV for 1) Moisture; 2) Starch; 3) Protein; 4) Lipids; 5) Fiber and 6) Ash.

**Figure S5**: Reference values ​​(Yref) versus predicted values ​​(Ypred) from the model calibration set preprocessed with MSC for 1) Moisture; 2) Starch; 3) Protein; 4) Lipids; 5) Fiber and 6) Ash.

**Figure S6**: Reference values ​​(Yref) versus predicted values ​​(Ypred) from the model calibration set preprocessed with SNV + BL + SSG for 1) Moisture; 2) Starch; 3) Protein; 4) Lipids; 5) Fiber and 6) Ash.

**Figure S7**: Reference values ​​(Yref) versus predicted values ​​(Ypred) from the model calibration set preprocessed with 1DSG for 1) Moisture; 2) Starch; 3) Protein; 4) Lipids; 5) Fiber and 6) Ash.

**Figure S8**: Reference values ​​(Yref) versus predicted values ​​(Ypred) from the held‐out test set without preprocessing for 1) Moisture; 2) Starch; 3) Protein; 4) Lipids; 5) Fiber and 6) Ash.

**Figure S9**: Reference values ​​(Yref) versus predicted values ​​(Ypred) from the held‐out test set with BL processing for 1) Moisture; 2) Starch; 3) Protein; 4) Lipids; 5) Fiber and 6) Ash.

**Figure S10**: Reference values ​​(Yref) versus predicted values ​​(Ypred) from the held‐out test set with SNV processing for 1) Moisture; 2) Starch; 3) Protein; 4) Lipids; 5) Fiber and 6) Ash.

**Figure S11**: Reference values ​​(Yref) versus predicted values ​​(Ypred) from the held‐out test set with MSC processing for 1) Moisture; 2) Starch; 3) Protein; 4) Lipids; 5) Fiber and 6) Ash.

**Figure S12**: Reference values ​​(Yref) versus predicted values ​​(Ypred) from the validation set of the model with SNV + BL + SSG processing for 1) Moisture; 2) Starch; 3) Protein; 4) Lipids; 5) Fiber and 6) Ash.

**Figure S13**: Reference values ​​(Yref) versus predicted values ​​(Ypred) from the held‐out test set with 1DSG processing for 1) Moisture; 2) Starch; 3) Protein; 4) Lipids; 5) Fiber and 6) Ash.

**Table S1**: Division of training and test sets by the Kennard‐Stone algorithm used in prediction

**Table S2**: Spectral absorption bands associated with components present in rice grains. Numbers related to specific bands are indicated in Fig. 6

**Table S3**: Statistical results of calibration and internal validation of partial least squares (PLS) regression models of parboiled rice samples, subjected to different spectral pretreatments (n=370).

**Table S4**: Statistical results of external validation of partial least squares (PLS) regression models of parboiled rice samples, subjected to different spectral pretreatments (n=124).

## Data Availability

Data will be made available on request.
