# Supplementary material for: Rapid Physicochemical Screening of Parboiled Rice Using VNIR–SWIR Spectroscopy and Chemometrics
Source: J Food Sci. 2026 Sep 30;91(10):e71501. doi: 10.1111/1750-3841.71501 (PMC13627962; doi:10.1111/1750-3841.71501)
Supplement: Supplementary file 1 [file JFDS-91-0-s011.docx]

**Table B.** Spectral absorption bands associated with components present in rice grains. Numbers related to specific bands are indicated in Fig. 7

| **Band (n°)** | **Spectral range (nm)** | **Average wavelength (nm)** | **Bond vibration** | **Component** | **Reference** |
| --- | --- | --- | --- | --- | --- |
| 1 | 835 - 900 | 867,5 | N-H (RNHR), third overtone and C-H | Protein and Amylose | LIU et al., 2020; TEYE E AMUAH (2022) |
| 2 | 900 - 945 | 922,5 | O-H | Water | TEYE E AMUAH (2022) |
| 3 | 985 | 985 | N-H stretching | Protein | WADOOD et al. (2019) |
| 4 | 1100 | 1100 | N-H e C-H-O | Protein and Starch | HAYATI et al. (2021) |
| 5 | 1200 | 1200 | C-H stretching | Lipids, Starch and Protein | WADOOD et al. (2019); WEI et al. (2020) |
| 6 | 1250 - 1275 | 1262,5 | C-H-O | Starch | HAYATI et al. (2021) |
| 7 | 1275 - 1315 | 1295 | C-H-O | Starch | HAYATI et al. (2021) |
| 8 | 1415 - 1515 | 1465 | C-O-N-H e O-H | Protein and Water | RACHMAWATI et al. (2017); HAYATI et al. (2021) |
| 9 | 1470 – 1526 | 1498 | C-H-O | Starch | HAYATI et al. (2021) |
| 10 | 1516 - 1604 | 1560 | N-H stretching, first overtone | Protein | HAYATI et al. (2021) |
| 11 | 1689 | 1689 | N-H | Protein | HAYATI et al. (2021) |
| 12 | 1860 | 1860 | C-H and O-H, first overtone and flexion | Lipids | HAYATI et al. (2021) |
| 13 | 1900 - 1978 | 1939 | O-H, first overtone | Water | RACHMAWATI et al. (2017); HAYATI et al. (2021) |
| 14 | 2175 - 2200 | 2187,5 | C-H-O, first and second overtones | Starch | YANG et al. (2018); AWANTHI et al. (2019); ISLAM et al. (2021) |
