# Supplementary material for: Rapid Physicochemical Screening of Parboiled Rice Using VNIR–SWIR Spectroscopy and Chemometrics
Source: J Food Sci. 2026 Sep 30;91(10):e71501. doi: 10.1111/1750-3841.71501 (PMC13627962; doi:10.1111/1750-3841.71501)
Supplement: Supplementary file 2 [file JFDS-91-0-s012.docx]

**Table C.** Statistical results of calibration and internal validation of partial least squares (PLS) regression models of parboiled rice samples, subjected to different spectral pretreatments (n=370). BL= Baseline Shift, SNV= Standard Normal Variation, MSC= Multiplicative Scattering Correction, SNV + BL + SSG Savitizky Golay Smoothing and 1DSG= 1st derivative Savitizky Golay

| **Component** | **Preprocessing** | **Calibration** | | | | **Internal (cross) validation** | | |
| --- | --- | --- | --- | --- | --- | --- | --- | --- |
|  |  | **Range (%)** | **R²** | **Correl** | **RMSEC** | **R²** | **Correl** | **RMSEVC** |
| Moisture | Without preprocessing | 11.19 -12.65 | **0.560** | **0.748** | **0.390** | **0.535** | **0.732** | **0.401** |
|  | BL |  | 0.554 | 0.744 | 0.393 | 0.522 | 0.722 | 0.407 |
|  | SNV |  | 0.557 | 0.746 | 0.392 | 0.534 | 0.729 | 0.403 |
|  | MSC |  | **0.562** | **0.750** | **0.389** | **0.539** | **0.733** | **0.400** |
|  | SNV + BL + SSG |  | 0.541 | 0.735 | 0.399 | 0.510 | 0.713 | 0.413 |
|  | 1DSG |  | 0.561 | 0.749 | 0.390 | 0.542 | 0.734 | 0.399 |
| Starch | Without preprocessing | 66.53 – 70.16 | **0.682** | **0.826** | **0.976** | **0.656** | **0.810** | **1.016** |
|  | BL |  | 0.674 | 0.821 | 0.989 | 0.648 | 0.805 | 1.028 |
|  | SNV |  | **0.680** | **0.824** | **0.980** | **0.655** | **0.808** | **1.020** |
|  | MSC |  | 0.673 | 0.820 | 0.990 | 0.647 | 0.803 | 1.031 |
|  | SNV + BL + SSG |  | 0.664 | 0.815 | 1.004 | 0.631 | 0.793 | 1.055 |
|  | 1DSG |  | 0.675 | 0.822 | 0.987 | 0.648 | 0.804 | 1.030 |
| Protein | Without preprocessing | 4.19 – 5.25 | 0.151 | 0.389 | 0.526 | 0.111 | 0.333 | 0.539 |
|  | BL |  | 0.155 | 0.393 | 0.525 | 0.114 | 0.336 | 0.539 |
|  | SNV |  | **0.165** | **0.407** | **0.522** | **0.126** | **0.353** | **0.535** |
|  | MSC |  | **0.166** | **0.408** | **0.521** | **0.127** | **0.353** | **0.535** |
|  | SNV + BL + SSG |  | 0.156 | 0.395 | 0.525 | 0.117 | 0.340 | 0.538 |
|  | 1DSG |  | 0.153 | 0.391 | 0.526 | 0.126 | 0.351 | 0.535 |
| Lipids | Without preprocessing | 1.44 – 1.96 | 0.462 | 0.680 | 0.191 | 0.436 | 0.660 | 0.196 |
|  | BL |  | 0.467 | 0.683 | 0.190 | 0.443 | 0.663 | 0.195 |
|  | SNV |  | **0.488** | **0.698** | **0.186** | **0.469** | **0.683** | **0.190** |
|  | MSC |  | **0.481** | **0.693** | **0.188** | **0.461** | **0.677** | **0.192** |
|  | SNV + BL + SSG |  | 0.472 | 0.687 | 0.189 | 0.445 | 0.665 | 0.194 |
|  | 1DSG |  | 0.466 | 0.683 | 0.190 | 0.447 | 0.666 | 0.194 |
| Fiber | Without preprocessing | 2.68 - 3.45 | 0.311 | 0.558 | 0.269 | 0.281 | 0.527 | 0.276 |
|  | BL |  | 0.326 | 0.571 | 0.266 | 0.296 | 0.541 | 0.273 |
|  | SNV |  | 0.353 | 0.594 | 0.261 | 0.326 | 0.568 | 0.267 |
|  | MSC |  | **0.360** | **0.600** | **0.259** | **0.333** | **0.574** | **0.266** |
|  | SNV + BL + SSG |  | 0.317 | 0.563 | 0.268 | 0.286 | 0.532 | 0.275 |
|  | 1DSG |  | 0.329 | 0.574 | 0.266 | 0.293 | 0.539 | 0.273 |
| Ash | Without preprocessing | 1.22 - 1.33 | **0.450** | **0.670** | **0.049** | **0.425** | **0.650** | **0.051** |
|  | BL |  | 0.444 | 0.666 | 0.050 | 0.422 | 0.647 | 0.051 |
|  | SNV |  | 0.447 | 0.668 | 0.049 | 0.420 | 0.735 | 0.051 |
|  | MSC |  | **0.450** | **0.670** | **0.049** | **0.425** | **0.650** | **0.051** |
|  | SNV + BL + SSG |  | 0.442 | 0.664 | 0.050 | 0.416 | 0.643 | 0.051 |
|  | 1DSG |  | 0.436 | 0.660 | 0.050 | 0.418 | 0.644 | 0.051 |

CP = Number of latent variables (components) used in the PLS model; Correl = Pearson correlation coefficient between predicted and reference values; R² = Coefficient of determination; RMSEC = Root Mean Square of Calibration.; RMSEVC = Root Mean Square Error of Cross Validation.
