# Supplementary material for: Rapid Physicochemical Screening of Parboiled Rice Using VNIR–SWIR Spectroscopy and Chemometrics
Source: J Food Sci. 2026 Sep 30;91(10):e71501. doi: 10.1111/1750-3841.71501 (PMC13627962; doi:10.1111/1750-3841.71501)
Supplement: Supplementary file 3 [file JFDS-91-0-s013.docx]

**Table D.** Statistical results of external validation of partial least squares (PLS) regression models of parboiled rice samples, subjected to different spectral pretreatments (n=124). BL= Baseline Shift, SNV= Standard Normal Variation, MSC= Multiplicative Scattering Correction, SNV + BL + SSG Savitizky Golay Smoothing and 1DSG= 1st derivative Savitizky Golay

| **Component** | Preprocessing | **CP** | **Range (%)** | **R²** | **Correl** | **RMSEP** |
| --- | --- | --- | --- | --- | --- | --- |
| Moisture | Without preprocessing | 4 | 11.19 -12.65 | **0.627** | **0.792** | **0.366** |
|  | BL | 4 |  | 0.622 | 0.789 | 0.369 |
|  | SNV | 2 |  | 0.615 | 0.785 | 0.372 |
|  | MSC | 2 |  | 0.621 | 0.788 | 0.369 |
|  | SNV + BL + SSG | 4 |  | 0.620 | 0.789 | 0.370 |
|  | 1DSG | 4 |  | - | -0.163 | 1.321 |
| Starch | Without preprocessing | 4 | 66.53 - 70.16 | 0.732 | 0.856 | 0.917 |
|  | BL | 4 |  | **0.740** | **0.861** | **0.902** |
|  | SNV | 2 |  | 0.724 | 0.851 | 0.930 |
|  | MSC | 2 |  | 0.724 | 0.851 | 0.929 |
|  | SNV + BL + SSG | 4 |  | 0.728 | 0.854 | 0.922 |
|  | 1DSG | 4 |  | - | -0.117 | 3.821 |
| Protein | Without preprocessing | 4 | 4.19 - 5.25 | 0.086 | 0.315 | 0.531 |
|  | BL | 4 |  | 0.084 | 0.307 | 0.531 |
|  | SNV | 2 |  | 0.055 | 0.290 | 0.540 |
|  | MSC | 2 |  | 0.059 | 0.296 | 0.539 |
|  | SNV + BL + SSG | 4 |  | **0.112** | **0.342** | **0.523** |
|  | 1DSG | 4 |  | - | -0.111 | 0.749 |
| Lipids | Without preprocessing | 4 | 1.44 - 1.96 | 0.557 | 0.754 | 0.173 |
|  | BL | 4 |  | 0.563 | 0.758 | 0.172 |
|  | SNV | 2 |  | **0.586** | **0.773** | **0.168** |
|  | MSC | 2 |  | **0.584** | **0.774** | **0.168** |
|  | SNV + BL + SSG | 4 |  | 0.555 | 0.753 | 0.174 |
|  | 1DSG | 4 |  | - | 0.004 | 0.350 |
| Fiber | Without preprocessing | 4 | 2.68 - 3.45 | 0.185 | 0.433 | 0.252 |
|  | BL | 4 |  | 0.204 | 0.453 | 0.249 |
|  | SNV | 2 |  | 0.232 | 0.485 | 0.244 |
|  | MSC | 2 |  | **0.243** | **0.496** | **0.243** |
|  | SNV + BL + SSG | 4 |  | **0.248** | **0.462** | **0.248** |
|  | 1DSG | 4 |  | - | -0.127 | 0.315 |
| Ash | Without preprocessing | 4 | 1.22 - 1.33 | 0.469 | 0.688 | 0.046 |
|  | BL | 4 |  | 0.470 | 0.687 | 0.046 |
|  | SNV | 2 |  | 0.480 | 0.696 | 0.045 |
|  | MSC | 2 |  | 0.482 | 0.697 | 0.045 |
|  | SNV + BL + SSG | 4 |  | **0.492** | **0.702** | **0.045** |
|  | 1DSG | 4 |  | - | -0.136 | 0.102 |

CP = Number of latent variables (components) used in the PLS model; Correl = Pearson correlation coefficient between predicted and reference values; R² = Coefficient of determination; RMSEP = Root Mean Square Error of Prediction.
