# Supplementary material for: Rapid Physicochemical Screening of Parboiled Rice Using VNIR–SWIR Spectroscopy and Chemometrics
Source: J Food Sci. 2026 Sep 30;91(10):e71501. doi: 10.1111/1750-3841.71501 (PMC13627962; doi:10.1111/1750-3841.71501)
Supplement: Supplementary file 4 [file JFDS-91-0-s016.docx]

**Table A.** Division of training and test sets by the Kennard-Stone algorithm used in prediction

| **Types** | **Sample** | | |
| --- | --- | --- | --- |
|  | *Treining* | *Test* | *Total* |
| Type 1 | 69 (62,79%) | 22 (20,02%) | 91 (100%) |
| Type 2 | 55 (47,3%) | 30 (25,8%) | 86 (100%) |
| Type 3 | 53 (39,75%) | 22 (16,5%) | 75 (100%) |
| Type 4 | 65 (56,55%) | 22 (19,14%) | 87 (100%) |
| Type 5 | 52 (34,84%) | 15 (10,05%) | 67 (100%) |
| Off-type | 76 (67,64%) | 13 (11,57%) | 89 (100%) |
